# Er:YAG Laser: A New Technical Approach to Remove Torus Palatinus and Torus Mandibularis

**DOI:** 10.1155/2012/487802

**Published:** 2012-06-27

**Authors:** J. P. Rocca, H. Raybaud, E. Merigo, P. Vescovi, C. Fornaini

**Affiliations:** ^1^Faculty of Odontology, University Hospital “St. Roch”, University of Nice-Sophia Antipolis, 5, rue Pierre Dévoluy, 06006 Nice, France; ^2^Oral Medicine and Laser-Assisted Surgery Unit, Faculty of Medicine, University of Parma, Viale Antonio Gramsci, 14, 43126 Parma, Italy

## Abstract

*Objective.* The aim of this study was to assess the ability of Er:YAG laser to remove by excision torus mandibularis and to smooth torus palatinus exostosis. *Materials and Methods.* Torus mandibularis (TM) and torus palatinus (TP) were surgically eliminated via the Er:YAG laser using the following parameters: TM: output power ranging from 500 to 1000 mJ, frequency from 20 to 30 Hz, sapphire tips (diameter 0.8 mm), air-water spray (ratio 5/5), pulse duration 150 **μ**sec, fluence ranging from 99592 J/cm^2^ to 199044,586 J/cm^2^. TP: a peeling technique was used to eliminate TP, as excision by slicing being impossible here. *Results.* TM: excision was obtained after 12730 pulses. TP: smoothing technique took more time compared with excision. Once peeling was considered to be accomplished, the use of a surgical rasp was necessary to eliminate bone spicules that could delay the wound to heal in good conditions. *Conclusion.* Er:YAG excision (TM) or Er:YAG peeling (TP) are safe clinical techniques easy to practice even if the time required for excision or surface smoothing is more than the time required with bony burs and high speed instruments.

## 1. Introduction

Tori may be considered as specific exostosis, formed by a highly dense and strictly limited amount of bone marrow, covered with a thin mucosa, easy to flap and poorly vascularised.

Their growth is very slow and do not produce any symptoms except in edentulous patients where constructing and wearing partial dentures seems hazardous to impossible.

The aetiology of tori is not clear at all [1] even if genetics is supposed to be the most widely accepted factor [2, 3]. Other causes such as functional responses to superficial injuries, temporomandibular disorders, eating habits and diet, vitamin deficiency, and drugs causing an increase in calcium homeostasis have been evoked. [4] On the other hand, some studies have been published on tori prevalence but conclusions did not demonstrate possible links between ethnical factors and aetiology [5].

Clinically, discovering of tori is frequently diagnosed in occasional way because those pathologies are asymptomatic. The request for clinical examination depends mainly on the size: in fact, in this case, they may perturb phonation, create ulceration of the mucosa, prosthetic instability or pain.

Conventional surgical treatment, in exception of chisel and hammers that involve possible risks of traumatic injuries, request to perform excision via bony burs once the flap has been anchored by different methodologies or simply elevated and maintained via suture needle or any other conventional means.

The aim of this paper is to demonstrate that Er:YAG laser may be an effective help in the surgical treatment of bony protuberances arising from cortical plate (torus palatinus, torus mandibularis), and that it may conducted rapidly and safely without potential damages to the surrounding tissues.

## 2. Cases Presentation

### 2.1. Torus Mandibularis Er:YAG Laser Removal

**Figure 1:**
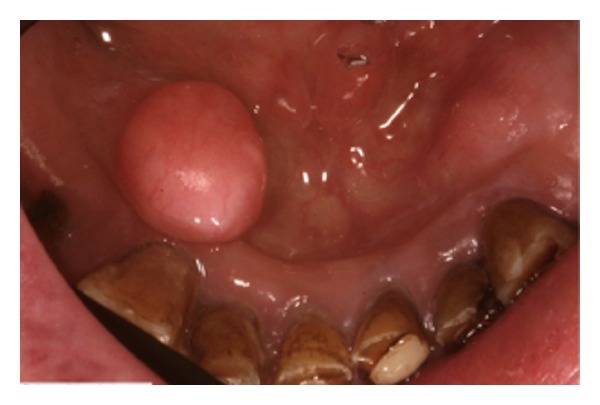
TM. preoperative view.

A 59-years-old male was referred to the clinic (Laser Unit, Pôle Odontologique, Centre Hospitalier Universitaire St Roch, Nice, France) for evaluation and treatment. The patient was concerned about an oral rehabilitation (partial denture) and the Department of Prosthetics asked for the removal of a large, round, lobular osseous protuberance (Figure 1) located in front of the buccal side of the mandibular premolars (teeth 44, 45). 

**Figure 2:**
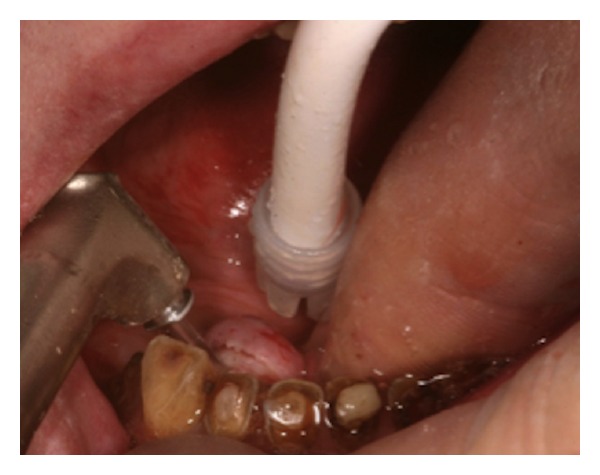
TM. Mucosal incision (sapphire tip).

This bony exostosis was covered with normal thin mucosa and the patient did not mention any identified symptoms. All the missing teeth (46, 47, 35, 36, 37) had to be replaced by a partial denture after carious decays plus root canal treatments and ceramic-metallic crowns aiming to serve the stability and the retention of the future prosthesis. General and oral health of the patient was satisfactory. Being clearly informed on the protocol to be engaged, the surgical procedure was performed. Local anaesthesia was delivered (direct infiltration in the mucosa with a short needle, 4% articaine). Er:YAG laser (Fotona Fidelis plus III, Slovenia) was used in respect of the following parameters: output power 500 to 1000 mJ, sapphire tip diameter 0.8 mm, pulse duration 150 *μ*sec, and fluence ranging from 99592 J/cm^2^ to 199044,586 J/cm^2^. Incision of the mucosa was performed with the lowest fluence (Figure 2) and rapidly obtained (pulses number 750 i.e., 750 × 150.10^−6^ sec = 112500 × 10^−6^ sec = 0.11 sec firing time not considering resting time, clinical working time 50 sec).

The flap was then removed and maintained via a metallic surgical spacer. In those conditions, the mouth floor was protected form laser hazards, being Er:YAG light totally reflected on metallic surfaces.

**Figure 3:**
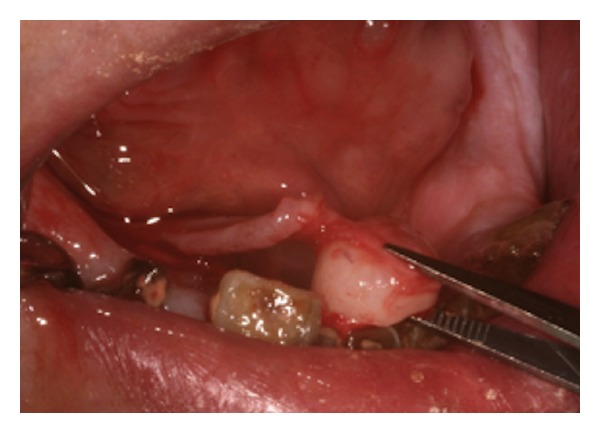
TM. Er:YAG laser excision.

Due to its gradual growth and its highly compact structure, high output power was used. The sapphire tip 1 mm far from the torus was used in a smooth linear movement close to the mandibular ridge and in pseudocontact of the TM base. The torus was completely sectioned (Figure 3) after 12730 pulses corresponding to a 1.9  sec laser light working time and a clinical working time of 5.37 minutes.

With Er:YAG laser being poorly absorbed in haemoglobin, the operative field is bleeding but at the same time washed with the air-water spray: subsequently, a high-powered aspiration is requested. Suturing was performed with 4.0 silk to let the wound heal by primary intention. The excised specimen was then placed in formaldehyde 10% for histopathological examination. An analgesic was immediately delivered to the patient (amidopyrin 500 mg). Some recommendations, such as to avoid any hot food or liquids during a 24 hrs long period were delivered. Sutures were removed one week later and complete healing observed after 12 days after surgery. Neither postoperative complications nor discomfort were observed. 

**Figure 4:**
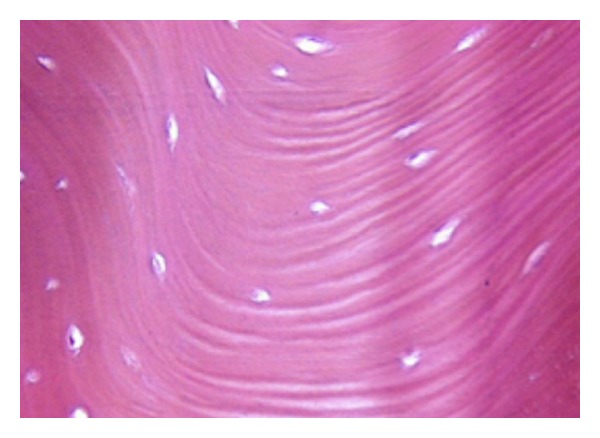
Photomicrograph of histological appearance of TM shows dense bony tissue, presence of lacunae and normal osteocytes (hematoxylin-eosin, original magnification 200x).

Hard tissue fragment was submitted in 10% formalin for histopathologic examination. The examination of hematoxylin and eosin staining specimen revealed (Figure 4) dense, mature bony tissue, organized in wide lamellar pattern with scattered osteocytes and small marrow spaces.

### 2.2. Torus Palatinus Er:YAG Laser Removal

**Figure 5:**
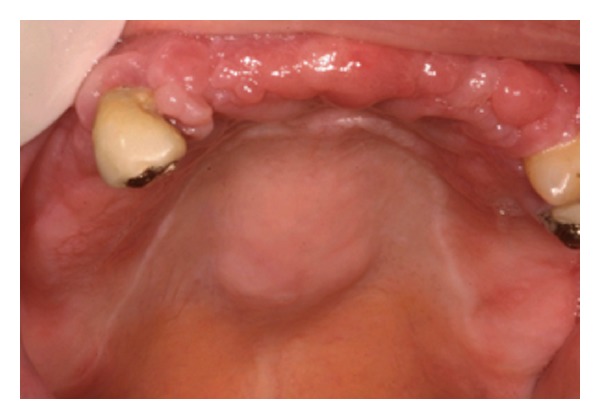
TP: aspect of the lesion before intervention.

A 67-years-old woman was referred to the Clinic for palatal bone exostosis removal (Figure 5).

**Figure 6:**
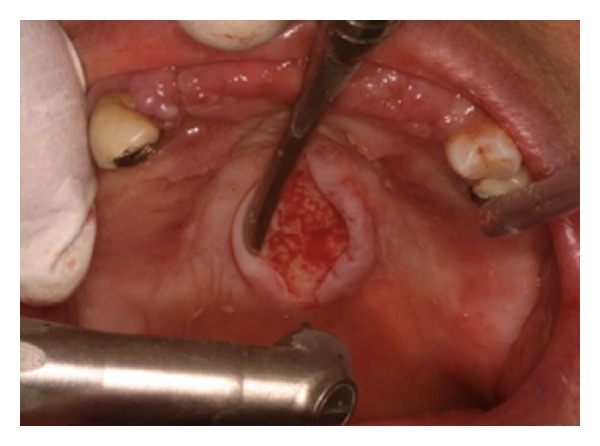
TP: the flap being removed, surface smoothing is engaged.

This exostosis covered the anterior region of the palatal vault without extension to the alveolar process. With this TP being poorly raised and in the same way large, excision by slicing or cutting was impossible whatever the technique used (bur or laser). It was possible to choose between two techniques: wearing away the TP with surgical burs or peeling/smoothing it with Er:YAG laser. It was decided to use Er:YAG laser the following parameters: output power 450 mJ, frequency 20 to 30 Hz, sapphire tip diameter 1.2 mm, pulse duration 150 *μ*sec, fluence 39808,91 J/cm^2^ air-water ratio 5/5, pulse number 12702 corresponding to (30 shots/sec) 421.4 sec that is, a little more than 7 minutes of laser working time. Local anaesthesia was delivered via infiltration of articaine 4%. Half thickness flap was easily tipped over (Figure 6) and the left side smoothed by firing. Same protocol was used for the right side. 

**Figure 7:**
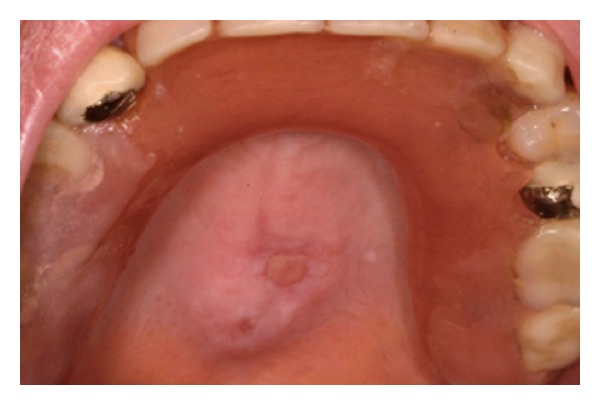
TP: suture being removed (7 days post-op), healing process is quite observed.

At the end of the surface treatment, a rasp was used to eliminate the possible remaining bony spicules. The suture was then made by simple points not too tight. Analgesic was immediately delivered and prescribed as previously described and the patient was informed that the signs and possible symptoms during the postoperative period might be those that are common with this type of surgical procedure. Moreover, she was informed and recommended to continue with appropriate hygiene. After one week, sutures were removed and the wound healed in good conditions (Figure 7). 

Due to the mechanism of tissue elimination with Er:YAG laser (explosive vaporization), it was impossible to take a sample for histopathological examination.

## 3. Discussion

Tori are bony swellings that develop slowly in the mouth. They are considered to be a developmental anomaly and they are classified according to their shape [6]:

- flat tori have a large base and are slightly convex with a smooth surface, generally symmetrical on to both sides of the mouth;
- spindle tori present as a midline ridge in the maxilla;
- lobular tori present as lobulated masses, arising from the single base;
- nodular tori arising as multiple protuberances with individual base.

The size of the tori may fluctuate throughout life and, when they interfere with function or partial/full denture placement, surgery is requested. However, in exception of suffering from recurring traumatic surface ulceration or mucosal problems or when contributing to a periodontal problem, removal of the tori is unnecessary. There is no report on possible malignant potential transformation [7, 8].

A lot of speculations have been reported on possible etiopathogenic processes even if the most widely accepted hypothesis is genetics [9–11]. However, it has not always been possible to demonstrate the autosomal dominant nature of its appearance. Prevalence of frequency (TP versus TM) is controversial too [12] as well as possible dominant sex group and ethnic groups [13, 14].

Tori are easily diagnosed by clinical examination. Usually the finding is incidental probably because they are asymptomatic for the patient even if some rare complains are reported.

While histopathological examination of TM shows a compact structure, TP microscopic structure is impossible to examine because they are neither nodular nor spindle but generally flat. Subsequently, surgery is conducted by remodelling the surface via bone-burr plus air-water spray. Er:YAG laser also remodels the surface via the so-called explosive vaporization of the target tissue. Each shot (pulse) takes of a small amount of bone and the repetition rate as well as the pulse duration, the spot size diameter, and the fluence are related to the efficiency of laser remodelling. As a consequence of a larger spot size, the energy delivered on the target tissue is reduced, fluence being expressed in Joules per centimetre square. Er:YAG laser tori removal, specifically for TP, takes more time than conventional methods. A little problem encountered in peeling the surface with this device regards the irregular surface observed once the TP has been Er:YAG treated: in fact, an irregular surface is present and it is related to the overlapping of the shots. For example, if 300 shots were delivered on a 1 mm^2^ area and only 100 shots are fired close to this treated surface, the amount of vaporized tissue is different and the surface, as a consequence, becomes irregular. For this reason, the use of a surgical rasp in order to prevent possible soft tissue damages is necessary before suturing the flap. 

Postoperative prescription and recommendations were identical to those previously described (TM).

## 4. Conclusion

Er:YAG laser is an optimal instrument to excise (TM) or smooth (TP) these lesions even if the time required for the intervention is more than the time needed by bony burs and high speed instruments.

Good clinical healing process obtained with this wavelength could be related to the reduction of target tissue heating, the decontamination, the absence of smear layer production that could disrupt the healing process, plus the biostimulation of the irradiated tissues.
